# Supplementary material for: Evidence supported by Mendelian randomization: impact on inflammatory factors in knee osteoarthritis
Source: Front Med (Lausanne). 2024 May 28;11:1382836. doi: 10.3389/fmed.2024.1382836 (PMC11165061; doi:10.3389/fmed.2024.1382836)
Supplement: Supplementary file 1 [file Data_Sheet_1.ZIP › data/GCST90274817_5e6/817.forest.pdf]

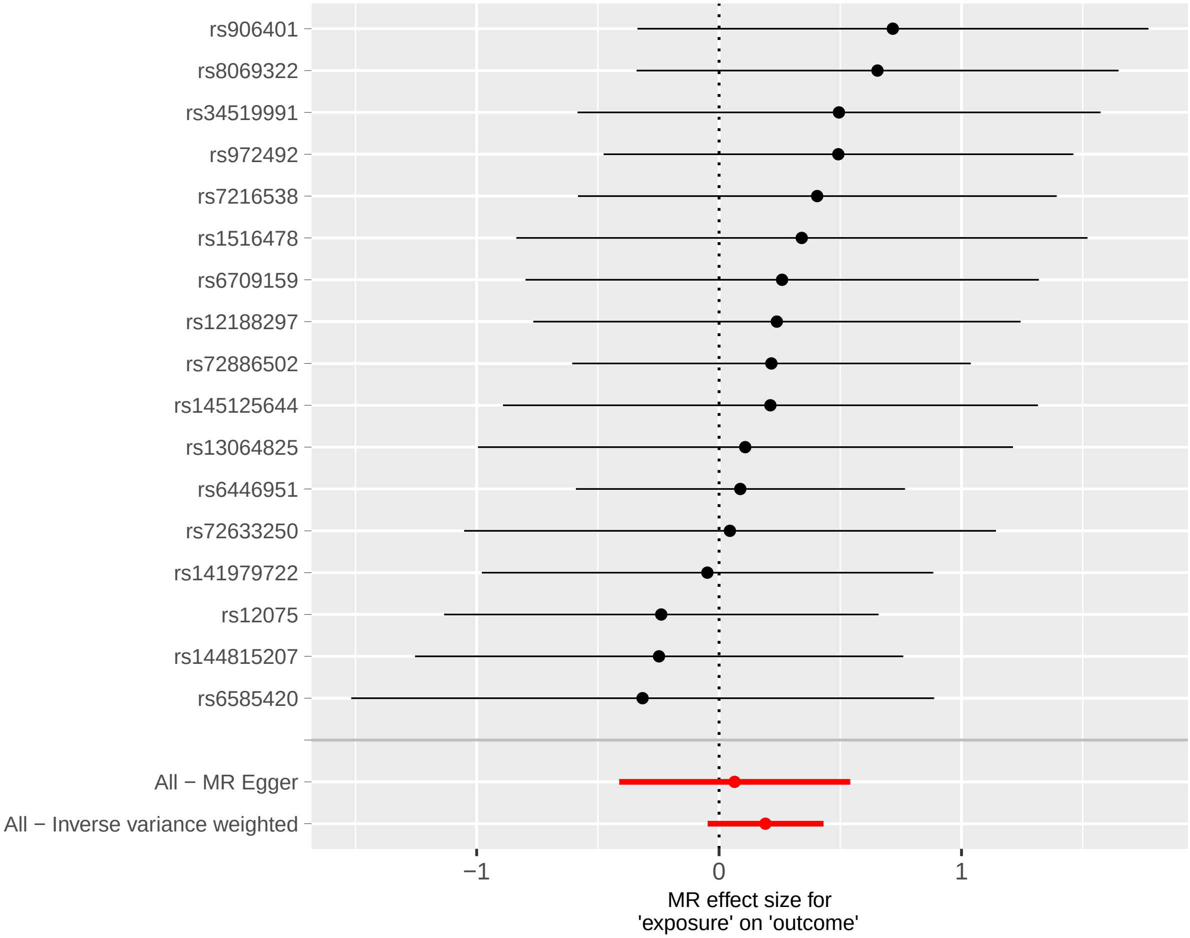
rs906401
rs8069322
rs34519991
rs972492
rs7216538
rs1516478
rs6709159
rs12188297
rs72886502
rs145125644
rs13064825
rs6446951
rs72633250
rs141979722
rs12075
rs144815207
rs6585420
All − MR Egger
All − Inverse variance weighted
−1
0
1
MR effect size for
'exposure' on 'outcome'
